# Harvest time and soil-plant relationship effects on phytochemical constituency and biological activities of *psidium guajava* L. leaves

**DOI:** 10.1038/s41598-025-11323-0

**Published:** 2025-07-17

**Authors:** Amal M. El-Feky, Mohamed A. E. AbdelRahman

**Affiliations:** 1 Pharmacognosy Department, National Research Centre, 33 El Bohouth St. (Former El Tahrir St.), P.O. 12622, Dokki, Giza, Egypt; 2 Land Use Department, Division of Environmental Studies and Land Use, National Authority for Remote Sensing and Space Sciences (NARSS), Cairo, 11769 Egypt

**Keywords:** Guava leaves, Harvest time, Phytoconstituents, Antioxidant, Antidiabetic, Anti-inflammatory, Biochemistry, Climate sciences, Health care, Medical research

## Abstract

Guava (*Psidium guajava* L.) leaves are deemed promising reservoir of phytoconstituents, with their characteristics potentially influenced by the timing of harvest and the dynamics of soil-plant interactions. The study revealed varying concentrations of minerals and vitamins in guava leaves, predominantly featuring vitamins B and C. Assessment of pigments using HPLC revealed that guava leaves collected in March had higher pigment concentration (461.233 mg/100 g) than that collected in August (447.084 mg/100 g). Quantification of total phenolics in guava leaves collected in March and August resulted in measurements of 435.21 ± 0.17 mgGAE/g and 294.31 ± 0.14 mgGAE/g, respectively. HPLC analysis demonstrated a diverse array of phenolic and flavonoid compounds present in *Psidium guajava*, with greater abundance and concentration of phenolic and flavonoid compounds in the samples harvested in March compared to those collected in August. For biological evaluation, guava leaves harvested in March demonstrated strong scavenging effect on DPPH and ABTS radicals, and considerable inhibition of carbohydrate-metabolizing enzymes (α-amylase, α-glucosidase, and β-galactosidase) in a dose-dependent manner. Furthermore, the March-collected guava leaves exhibited notable inhibition of COX-2 and 5-LOX enzyme activities, surpassing the effects of leaves collected in August. The study’s outcomes demonstrate richness of phytoconstituents in guava leaves, which underpin various biological functions, particularly during spring relative to the summer. This highlights the importance of the timing of collection in assessing phytochemical properties and their biological implications, highlighting the necessity of considering this aspect when sampling guava leaves.

## Introduction

Guava (*Psidium guajava* L.), a member of the Myrtaceae family, is extensively cultivated and valued as a significant fruit in tropical regions such as India, Indonesia, and South America. This traditional plant is esteemed for its wide-ranging medicinal and nutritional benefits. The Egyptian Bassateen El Sabahia guava holds significant value in Egypt for its health-promoting and nutritional properties. The leaves of this guava are utilized in the treatment of gastrointestinal disorders, such as diarrhea, as well as respiratory conditions, owing to their antimicrobial characteristics. The fruit itself, abundant in Vitamin C and antioxidants, enhances immune function and is commonly consumed fresh or incorporated into various products, including juices and jams^1,2^. Various parts of the guava tree, including its leaves, stems, and fruits, have been utilized in numerous countries for the treatment of ailments such as stomach pain, diabetes, diarrhea, and other health issues^3^. *Psidium guajava* leaves are characterized by their dark green hue and elliptical to oval shape, featuring an obtuse apex. These leaves have been employed in the management of numerous respiratory and gastrointestinal conditions^4^. Additionally, they are widely acknowledged for their properties as antispasmodics, cough suppressants, anti-inflammatories, and agents for managing diabetes^5^. Recent studies conducted on animal models have demonstrated the potential of *P. guajava* leaves as effective antitumor, anticancer, and cytotoxic substances^6–8^. The bioactive and therapeutic characteristics of *P. guajava* leaves are largely attributed to a distinctive array of bioactive polyphenolic compounds, including quercetin, flavonoids, and various phenolic acids such as ferulic, caffeic, and gallic acids^9^. A multitude of studies highlight the advantageous effects of integrating *P. guajava* leaf extract into food products as a functional ingredient, due to the presence of numerous compounds like rutin, naringenin, gallic acid, catechin, epicatechin, kaempferol, isoflavonoids, and vitamins, all of which are recognized for their positive impacts on human health^10^.

The fluctuations in primary metabolic processes in plants across different seasons are well documented^11^. The production and accumulation of secondary metabolites in plants influenced by the stages of cultivation. Throughout the various growth phases, numerous enzymes are involved in the biosynthetic pathways of phenolic compounds. Additionally, environmental factors significantly impact these processes. Factors such as soil characteristics and cultivation practices can modify the expression of various enzymes that participate in phenolic biosynthesis, thereby affecting the interactions with plant secondary metabolites. These enzymes are subject to complex regulatory controls and demonstrate specific responses to environmental changes, particularly those related to seasonal variations^12^.

The characteristics of soil, including its nutrient composition, play a crucial role in shaping the phytochemical profile of plants. Soils that are rich in organic matters and essential nutrients are associated with an increased production of beneficial phytoconstituents^13^. Thus, both the optimal timing for harvest and the quality of soil are vital for optimizing the beneficial effects of the plant. Ignoring these factors can lead to erroneous or inconsistent evaluations of the potency and composition of phytochemical extracts. Therefore, to ensure a commercially reliable output of *P. guajava* leaves with consistent high quality and functionality, the evaluation of functional constituents, along with antioxidant, antidiabetic, and anti-inflammatory activities, was performed, in relation to harvest timing and the soil-plant interaction.

## Methodology

### Chemicals and reagents

All chemicals utilized in this study were of analytical grade. Standards and solvents used in the study were sourced from Merck and Co., Inc., USA and Sigma-Aldrich. TLC plates (analytical and preparative), were purchased from Sigma Aldrich, Darmstadt, Germany. Additionally, 1,1-Diphenyl-2-picryl-hydrazyl (DPPH) sourced from Aldrich Chemie in Germany, as well as 2,2’-azinobis-(3-ethylbenzothiazoline-6-sulfonic acid) (ABTS) from Fluka Biochemika, Sigma-Aldrich, were employed. Additionally, the carbohydrate-metabolizing enzymes, which include purified α-amylase, α-glucosidase, and β-galactosidase, were obtained from Sigma Chemical Company in New York, USA.

### Plant collection, authentication and extraction

Intensely green leaves of *Psidium guajava* “Egyptian Bassateen El Sabahia guava”, devoid of any diseases, insect infestations, or mechanical injuries, were harvested from a five-year-old tree located on a private farm in El-Salheya Elgdeda, Sharkia Governorate, Egypt. Permission and informed consent were obtained from the private farm owner before collecting the leaves of Psidium guajava for this study. The collection occurred just before the flowering phase in March and during the fruiting period in August 2024. During this timeframe, the environmental conditions were characterized by an average maximum temperature of 25 °C (77 °F) during the day and a minimum of 15 °C (59 °F) in the early mornings or evenings in spring. In August, daytime temperatures reached highs of 35 °C to 40 °C (95 °F to 104 °F), while nighttime lows ranged from 23 °C to 28 °C (73 °F to 82 °F). Precipitation levels varied from 0 to 0.6 mm, and the daily light saturation duration ranged between 8.45 and 10.30 h.

The taxonomical identification of the leaves was performed by Prof. Dr. Gamal Farag at the Horticulture Research Centre of the Ministry of Agriculture. A specimen was archived in the Herbarium of the National Research Centre in Egypt, where it has been assigned the registration number M259.

Leaves of *P. guajava* that were gathered underwent a gentle rinsing with distilled water to eliminate any dust particles. Following this, they were shade-dried and ground into a fine powder using a blender. To achieve a consistent particle size, the powder was subsequently passed through an aluminum sieve with a mesh size of 1 mm. The extraction of phenolic compounds was conducted in accordance with the methodology established by^14^. In summary, two separate batches of dried powdered *P. guajava* leaves, each weighing 350 g and collected in March and August, were extracted using a solvent mixture of ethanol and water in a 75:25 ratio (v/v) at room temperature, with a total volume of 3 L for six extractions. The extracts were then concentrated using a rotary evaporator (Heidolph, Germany) at a temperature of 50 °C, resulting in dark green residues weighing 46 g dry weight (13.4% W/V) and 43.5 g dry weight (12.43%W/V), respectively. The concentrated extracts were further defatted through partitioning with petroleum ether (300 ml, four times), yielding defatted residues of 7.3 g and 6.9 g dry weight, respectively. The remaining extracts were subjected to extraction with ethyl acetate, leading to the concentration of the phenolic-rich fractions, which provided dried residues of 19.5 g and 17.6 g dry weight, respectively, for subsequent phytochemical and biological evaluations.

### Soil sampling

Soil type criteria were defined to evaluate the influence of soil composition on the phytoconstituents of dried *P. guajava* leaves^15^. The collection of soil samples was facilitated through the use of a soil auger and compositing soil samples followed. Soil samples were obtained from 0 to 150 cm depth range. The soil samples were collected, air dried, sealed, and stored in a polypropylene container in which was analysed for soil properties and other physical and chemical analyses after which the characteristic tests were conducted. Saturation soil paste extract analyses which covered most of the methods specified in the soil survey laboratory methods handbook (USDA, 2004) customs were made of the samples. These included soil texture, CaCO3 content, salinity EC, sodicity pH, soluble cations and anions, organic matter, and gypsum content analyses. Samples were first crushed and then examined through a set of sieves 2 mm mesh size first, then 0.5 mesh thereafter the powders were left to dry at room temperature while analysing the mid-infrared diffused reflected spectrum.

Soil samples were tested for TN, P and S, exchangeable cations (Ca, Mg, Na and K), soil micronutrients (Fe, Cu, Mn, Zn, and B), and cation exchange capacity (CEC). The Mehlich-3 multi-nutrient extraction method (1984) was also used to determine some of these parameters in soil samples. An Inductively coupled plasma (ICP) spectrometer was utilized to quantify the concentration of the extracted components. Using MIR-diffused reflectance spectral analysis, TAN and CEC were also measured.

### Assessment of disease incidence

The determination of disease incidence in *P. guajava* leaves, collected in the months of March and August, was performed by employing the formula specified by Iqra et al.^16^: Disease Incidence (D.I.) = Number of diseased leaves / Total number of examined leaves × 100.

### Phytochemical constituency

#### Macro and micro‑elements in *P. guajava* L. leaves

The nutritional composition of dried *P. guajava* leaves, collected in March and August, was assessed separately for both macro and microelements. The analysis encompassed macroelements such as Phosphorus, Potassium, Calcium, Sodium, and Magnesium, alongside microelements including Zinc, Copper, Iron, and Manganese. The concentrations of Potassium, Calcium, Sodium, and Magnesium, as well as the microelements, were measured using an atomic absorption spectrophotometer (Perkin Elmer, 1100B), adhering to the methodology proposed by Aboulthana et al.^17^. Furthermore, the Phosphorus content was evaluated using a spectrophotometer (Perkin-Elmer Lambda 2) in accordance with the vanado-molybdate method established by Jackson^18^.

#### Vitamins contents in *P. guajava* leaves

The quantification of vitamins in dried *P. guajava* leaves, collected during the months of March and August, was conducted utilizing high-performance liquid chromatography (HPLC) with a Shimadzu-UFLC Prominence system. This system featured an ultraviolet-visible (UV-Vis) detector (Model-SPD 20 A), in accordance with the methodology established by Hasan et al.^19^. The analysis of the results was performed following the protocol detailed by Aboulthana et al.^20^.

#### HPLC analysis of natural pigments

The HPLC analysis procedures used to determine the levels of chlorophyll a, b, and β-carotene in the ethyl acetate fraction of dried *P. guajava* leaves, which were collected in March and August, were carried out according to the guidelines set by Siong and Lam^21^.

#### Quantifiable valuation of total phenolics

The total polyphenol concentration in the ethyl acetate fraction of dried *P. guajava* leaves, collected in March and August, was quantified as mg of gallic acid per 100 g. This was achieved using the Folin-Ciocalteu reagent, based on the method described by El-Feky et al.,^22^. The results were presented as equivalents of gallic acid.

#### Quantifiable valuation of total flavones and flavonols

The total flavones and flavonols in the ethyl acetate extract of dried *P. guajava leaves*, collected in March and August, were quantified as described by Bahloul et al.^23^ using 2% AlCl_3_-ethanol solution. Absorbance readings were taken at 420 nm, and the results were presented in terms of mg rutin equivalent.

#### Quantifiable valuation of total flavanones and dihydroflavonols

The total content of flavanones and dihydroflavonols in the ethyl acetate extract of dried *P. guajava* leaves, collected in March and August, was evaluated following the method outlined by Bahloul et al.^23^. The assessment utilized a 2,4-dinitrophenylhydrazine solution, and absorbance was measured at 486 nm, with results expressed as mg of naringenin equivalent.

#### HPLC identification of phenolics and flavonoids

High-performance liquid chromatography (HPLC) analysis was performed on the ethyl acetate extract of dried *P. guajava* leaves collected in March and August at the Central Labs in NRC, Cairo, Egypt. An Agilent 1260 series instrument equipped with a C18 column (4.6 mm x 250 mm i.d.) was used for the analysis. The mobile phase was a mixture of water (A) and 0.05% trifluoroacetic acid in acetonitrile (B), flowing at a rate of 0.9 ml/min. A linear gradient program was set with the following parameters: 0 min (82% A); 0–5 min (80% A); 5–8 min (60% A); 8–12 min (60% A); 12–15 min (82% A); 15–16 min (82% A); and 16–20 min (82% A). The analysis utilized a multi-wavelength detector set to 254, 280, 320, and 360 nm. The identification of phenolic compounds was accomplished by comparing their retention times with those of standard phenolics and flavonoids^24^.

#### Isolation of major flavonoids

The extracts from dried *P. guajava* leaves, collected in the months of March and August, were each processed through Preparative TLC chromatography on an Aluminum TLC plate coated with silica gel 60G and a fluorescent indicator F254 (Merck, dimensions 20 × 20 cm, thickness 250 μm). The chromatographic development employed a solvent system comprising ethyl acetate, dichloromethane, formic acid, acetic acid, and water in a ratio of 100:10:10:10:11 (v/v/v/v/v)^25^. The chromatograms, once developed and dried, were subjected to scanning in fluorescence mode at a wavelength of 365 nm, both before and after treatment with a 1% ethanolic solution of AlCl_3_. Bands that indicated a positive reaction to the spraying reagent were collected individually. The purification process continued on thin-layer chromatography (TLC) plates, employing a solvent system of methanol, chloroform, and hexane in a 7:2:1 (v/v/v) ratio, and the retention factor (R_*f*_) values for the distinct bands were subsequently determined^26^. Flavonoid-containing bands were carefully excised with surgical blades, dissolved in ethanol, and subsequently filtered using Whatman No. 42 filter paper. The structural elucidation of the isolated compounds was conducted through Nuclear Magnetic Resonance (NMR) spectroscopy using JEOL instruments at frequencies of 270 MHz, 400 MHz, and 125 MHz for the respective analyses of ^1^H-NMR and ^13^C-NMR.

### Biological investigation

#### Free radical scavenging assay

Two distinct antioxidant assays were performed to evaluate the radical scavenging potential of the ethyl acetate fraction obtained from dried *P. guajava* leaves collected in March and August. The assays involved the scavenging of DPPH and ABTS radicals at various concentrations (10, 50, 100 µg), following the methods suggested by Rahman et al.^27^. Ascorbic acid served as the reference standard.

#### In vitro anti-diabetic activity

The anti-diabetic effects of the ethyl acetate fraction from dried *P. guajava* leaves, collected in March and August, were compared in vitro. This comparison involved measuring the inhibition of carbohydrate hydrolyzing enzymes, including α-amylase, α-glucosidase, and β-galactosidase, as detailed in Naguib et al.^28^.

#### In vitro antiinflammatory evaluation

The anti-inflammatory effects of the ethyl acetate fraction of dried *P. guajava* leaves, collected in March and August, were evaluated in vitro using different concentrations (1,10,100 µg). This evaluation involved measuring the inhibition of COX-2 using Inhibitor Screening Kits (Milpitas, CA, USA), with absorbance readings taken at UV-410 nm alongside a blank. Indomethacin was used as a reference drug for this assay. Furthermore, an inhibition assay was performed with the 5-LOX enzyme (human recombinant) using a 5-LOX kit (Sigma-Aldrich), and results were compared to those obtained with Zileuton as a reference drug. Absorbance was measured at UV-490 nm against a blank. The assay methodology was based on the work of El-Feky and El-Rashedy^29^. IC50 values were derived from the inhibition curves using the sigmoid dose-response model and linear regression analysis in Microsoft Office Excel 2010.

### Statistical analyses

All experiments were conducted three times, and the data is presented as mean ± standard deviation. Statistical analysis was carried out using one-way analysis of variance (one-way ANOVA) with the Statistical Package for Social Sciences (SPSS for Windows, version 11.0).

## Results and discussion

### Soil characterization

In the area studied, texture and CEC are vital components wherein crop could acquire information on nutrients and water in the root zone. Results represented further Depict these features of the soil sample that had been examined. Moreover, the presence of soil texture is important in maintaining soil moisture, which is helpful during the growing period of crop and also in connection with the existing irrigation practices. However, the soil is homogenous in area and from the surface and subsurface layers and/or horizons^30^. The physical and chemical properties of the soil were conducted, and the findings are detailed in Tables 1 and 2. Soil pH, value of 7.9. Therefore, it is considered neutral to moderately alkaline. There were no appreciable variations between horizons. The pH of the soil is 2.1 dS/m. Percent organic matter is 0.91%. Calcium carbonate is 1.5%. Alluvial deposit-growing soils contain very little. Mainly, the dominant soil textural types were clay loam in a relatively uniform pattern. Pedological analyses showed that agricultural and sustainable crop production in this region is ideal. For that reason, the water and minerals supplying to crops was considered acceptable; deep soils and good drainage offer the ability to have crops with good root development, thus a positive growth condition. Total N in soil comprises inorganic and organic nitrogen, the latter dominating. Plants, however, only utilize the inorganic fraction of N. This then implies that optimum TN could not be regarded as a guarantee with respect to plant productivity. Low levels of TN might be due to reduced soil OC content, insufficient and unbalanced fertilizer use, intensive cropping systems, and N losses through leaching^15,30^. Moreover, nutrient mining and low OM applications which were frequent in the studied area, could lead to a decline in TN level. High variability in P concentration might result in variation of soil management practices^31–36^. The obtained value showed a K deficiency. Hence, K fertilizer application should be implemented. The basic cations on the exchange site. The result showed in the order of Ca > Mg > K.

**Table 1:** The physical and chemical properties of the analyzed soil.

Sand(%)	Silt (%)	Clay(%)	Texture class	Organic matter %	CaCO_3_	pH	E.C. (dS/m)	ESP	CEC cmol(^+^)kg^− 1^
29.1	33.4	37.5	Clay- loam	0.9	1.5	7.9	2.1	4.5	25.13

**Table 2:** The macro- and micro-nutrients in the analyzed soil.

Total *N* mg/kg soil	Ca^2+^ mg/kg soil	K^+^ mg/kg soil	Na^+^ meq/l	Mg^2+^ mg/kg soil	*P* mg/kg soil	Cl^−^ meq/l	SO_4_^−^ meq/l	Zn mg/kg soil	Fe mg/kg soil	Cu mg/kg soil	Mn mg/kg soil	B mg/kg soil
105	343.4	379.6	11.5	201.3	3.6	16.8	35.14	0.86	23.4	2.37	94.94	0.79

### Disease incidence

The incidence of disease in *P. guajava* leaves harvested in March was 16.43 ± 1.25, compared to 21.37 ± 1.16 in August. This shows that *P. guajava* leaves are less affected by disease before flowering than they are during the fruiting stage.

### Macro and micro‑elements of *P. guajava* leaves

The macro and micro-element contents of dried *P. guajava* leaves collected at two different harvest times are shown in Table 3. There were variable concentrations of minerals in *P. guajava* L. leaves collected in March and August. The nitrogen content in dried *P. guajava* leaves from March and August was found to be notable, at 2.19% and 1.97%, respectively. Similar findings regarding nitrogen content in dried *P. guajava* leaves were reported by Tomar et al.^37^.

The levels of potassium and calcium in dried *P. guajava* leaves were found to be adequate. Specifically, the potassium concentration in leaves harvested in March and August was recorded at 1.96% and 1.38%, respectively. The potassium levels in the majority of dried *P. guajava* leaves fell within the optimal range. These findings are consistent with those reported by Tomar et al.^37^. Furthermore, the calcium content in *P. guajava* leaves collected during the same months was measured at 1.87% and 1.65%, respectively, with calcium being the most abundant mineral present. This suggests that guava leaves may serve as a beneficial dietary supplement for addressing calcium deficiencies, as noted by Gurusamy et al.^38^.

The magnesium levels in *P. guajava* leaves gathered in March and August were found to be moderate, measuring 0.53% and 0.47%, respectively. Additionally, phosphorus and sodium were present in reasonable quantities. Specifically, the phosphorus concentrations in the *P. guajava* leaves collected during these months were 0.31% for March and 0.27% for August, aligning with the findings of Singh and Singh^39^. In terms of sodium, the levels recorded were 0.24% in March and 0.19% in August.

In terms of micro-elements analysis, the iron levels in *P. guajava* leaves collected in March and August were determined to be 72.10 ppm and 56.13 ppm, respectively. The majority of these leaves contained iron concentrations that fell within the optimal range of 50–120 ppm, corroborating the findings of Parihar et al.^40^. Furthermore, Manganese content in dried *P. guajava* leaves for the same months was recorded at 43.62 ppm and 41.05 ppm, which aligns with the optimal range reported by Kotur et al.^41^. The copper levels in *P. guajava* leaves collected in March and August were found to be 12.50 ppm and 10.63 ppm, consistent with the observations of Singh and Singh^39^, who noted copper levels between 4.0 and 14.0 ppm dried *P. guajava* leaves. Lastly, the zinc concentrations in *P. guajava* leaves for March and August were the lowest at 9.76 ppm and 8.54 ppm, respectively, indicating a deficiency in zinc as highlighted by Tomar et al.^37^.

The study’s findings affirm that guava leaves are an abundant source of macro and micro-elements, positioning them as a highly beneficial option for human nutrition and as an effective animal feed to address micronutrient deficiencies^42^. The calcium-rich nature of dried *P. guajava* leaves may assist in reducing the likelihood of deficiency-related ailments such as hypocalcemia and osteoporosis^43^. A daily consumption of 100 g of dried guava leaves, whether prepared as tea or taken in capsule form, can yield 34.85%, 79.34%, and 46.11% of the recommended daily intake for potassium, iron, and zinc, respectively, which is greater than the amounts provided by the fruit^44^.

Another notice that the levels of macro and microelements in dried *P. guajava* leaves exhibit considerable variation, which can be attributed to the physicochemical characteristics of the soil, the fertility status, and various agricultural practices, resulting in differing nutrient profiles across plantations^45^. Nitrogen and potassium, due to their mobile nature, are translocated from older leaves to the meristematic tissues, with peak levels observed in March, followed by a decline from April until the fruit harvesting period.

The observed spike in foliar nitrogen and potassium levels in March may be attributed to the emergence of new leaves and the mobilization of nutrients from storage tissues to active growth sites, with 63–77% of the nitrogen required for spring growth and flowering sourced from the tree’s reserves. Developing fruits act as sinks for nitrogen, phosphorus, and potassium, leading to the mobilization of these nutrients from the leaves to the fruits, which explains the decreasing trend of these elements during August and September^46^. Consequently, it is recommended that *P. guajava* leaves be collected prior to flowering in March to ensure a high mineral concentration.

**Table 3:** Macro and micro-elements of dried *P. guajava* leaves, which collected in March and august.

Collected guava leaves	Macro‑elements (%)						Micro‑elements (ppm)			
	*N*	K	Ca	Mg	*P*	Na	Fe	Mn	Cu	Zn
**In March**	2.19	1.96	1.87	0.53	0.31	0.24	72.10	43.62	12.50	9.76
**In August**	1.97	1.38	1.65	0.47	0.27	0.19	56.13	41.05	10.63	8.54

### **Vitamins contents in** dried *P. guajava* leaves

The vitamin content in dried *P. guajava* leaves, collected during two different times of the year, is presented in Table 4. The results demonstrated varying concentrations of vitamins B and C in *P. guajava* L. leaves harvested in March compared to those collected in August, with the March samples exhibiting superior concentrations than those from August. This evidence underscores the notion that the seasonal timing of plant harvesting can significantly influence the nutritional quality of the leaves.

In the current study, the concentrations of vitamins B1, B2, B3, and B6 in dried *P. guajava* leaves collected in March were found to be 18.24, 29.43, 13.65, and 9.76 mg/100 g, respectively, surpassing the levels recorded in August, which were 15.07, 24.11, 9.63, and 7.85 mg/100 g. Furthermore, the Vitamin C concentration in the March samples was notably high at 96.42 mg/100 g, compared to 87.15 mg/100 g in August. These results are consistent with the findings of Thomas et al.^47^. The data obtained from this investigation indicate that guava leaves represent a significant source of vitamins B and C, thereby validating their diverse therapeutic uses in folk medicine, as highlighted by Pandian and Jayalakshmi^48^. It has been established that vitamin B is crucial for enhancing blood circulation, facilitating nerve relaxation, and stimulating cognitive functions, while the higher vitamin C content in dried *P. guajava* leaves may contribute to immune system enhancement and the maintenance of healthy blood vessels^43^.

**Table 4:** Vitamins contents of dried *P. guajava* leaves, which collected in March and august.

Collected guava leaves	Concentration (mg/100 g)				
	B1	B2	B3	B6	C
**In March**	18.24	29.43	13.65	9.76	96.42
**In August**	15.07	24.11	9.63	7.85	87.15

### HPLC analysis of natural pigments

The pigment profile of the ethyl acetate fraction from dried *P. guajava* leaves collected in March and August was evaluated through HPLC. The values for Chlorophyll a, b, β-Carotene, and the total identified pigments are summarized in Table 5. The analysis demonstrated that the dried *P. guajava* leaves which collected in March had a higher pigment content (461.233 mg/100 g) compared to those collected in August (447.084 mg/100 g). In March, the concentrations of chlorophyll a, b, and β-Carotene were found to be 167.039, 293.251, and 0.943 mg/100 g, respectively, whereas the August samples showed chlorophyll a, b, and β-Carotene levels of 159.264, 287.034, and 0.786 mg/100 g.

The fluctuations in the abundance and composition of pigments were influenced by the timing of the harvest. Gaining insight into the temporal variations in phytochemical composition is essential for elucidating the regulatory mechanisms involved and ultimately enhancing the quality of phytochemical products.

**Table 5:** Natural pigments identified from the Ethyl acetate fraction of dried *P. guajava* leaves which collected in March and august.

Collected guava leaves	Concentration (mg /100 g dry weight)			
	Chlorophyll a	Chlorophyll b	β-Carotene	Total identified pigments
**In March**	167.039	293.251	0.943	461.233
**In August**	159.264	287.034	0.786	447.084

### Quantifiable valuation of total phenolics and flavonoids

**Fig. 1:**
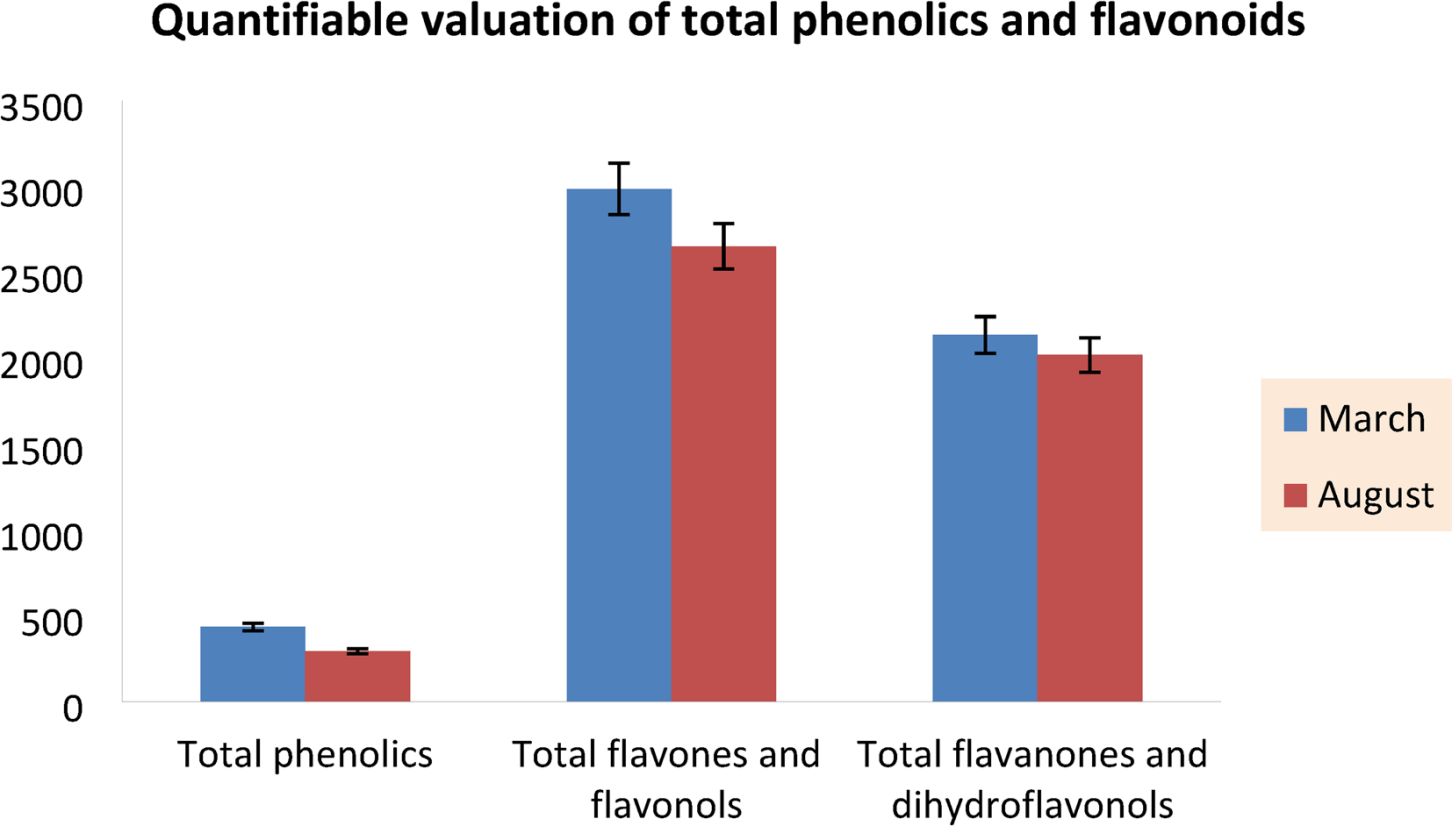
The quantification of total phenolics and flavonoids in the ethyl acetate fraction of dried *P. guajava* leaves, collected during March and August.

The assessment of total phenolics and flavonoids in the ethyl acetate fraction of dried *P. guajava* leaves, gathered in March and August, was performed, with the comprehensive results illustrated in Fig. 1. The total phenolic content in the ethyl acetate fraction of dried *P. guajava* leaves collected in March and August was found to be 435.21 ± 0.17 mg GAE/g and 294.31 ± 0.14 mg GAE/g, respectively. The analysis also indicated that the flavones and flavonols were present at concentrations of 2986.13 ± 0.17 mg rutin equivalent/100 g fraction and 2651.40 ± 0.14 mg rutin equivalent/100 g fraction, for the months of March and August, respectively. However, the concentrations of flavanones and dihydroflavonols were significantly lower, recorded at 2135.17 ± 0.12 mg naringenin equivalent/100 g fraction and 2018.07 ± 0.10 mg naringenin equivalent/100 g fraction, respectively, for the months of March and August.

The results of this study substantiate the notable concentration of total phenolics and flavonoids in dried *P. guajava* leaves, which underpins various biological functions, especially during the spring season prior to flowering in March, as compared to their levels during the fruiting phase in August. These findings align with the research conducted by Qian and Nihorimbere^49^.

The observed variation in phenolic content in the ethyl acetate fraction from dried *P. guajava* leaves, which collected in March and August may be linked to climatic conditions. The high temperatures prevalent in August could potentially lead to the degradation and decomposition of certain thermolabile phenolic and flavonoid compounds. Phenolic compounds, which exhibit a range of chemical structures, typically contain at least one aromatic ring, with one or more hydrogen atoms replaced by hydroxyl groups. These compounds are recognized for their diverse biological effects, which include anti-inflammatory, antimicrobial, hypolipidemic, anticancer activities, as well as antioxidant properties^50,51^.

### Phenolic and flavonoids identification by HPLC

Phenolic and flavonoid compounds are pivotal bioactive constituents that contribute significantly to the management of various metabolic and physiological functions in the human body^52^. The increasing interest in bioactive compound-rich diets in recent years can be attributed to their potential to decrease the risk of numerous chronic diseases^53,54^. The impact of agronomic and environmental conditions on the levels of phenolic compounds in plants and their bioactivities has been extensively discussed in the literature^55,56^. As a result, this evaluation seeks to explore the variability of phenolic and flavonoid compounds in *P. guajava* leaves across different harvesting times.

The HPLC analysis conducted on the ethyl acetate fractions of dried *P. guajava* leaves collected in March and August demonstrated a diverse array of phenolic and flavonoid compounds present during both harvesting periods. Specifically, 15 distinct compounds were identified in the March samples, whereas 3 compounds were detected in the August samples. Comprehensive results are available in the accompanying Table 6.

Among the compounds detected in the ethyl acetate fraction of *P. guajava* leaves gathered in March, syringic acid (0.43 mg/g) and methyl gallate (0.32 mg/g) were identified as the main phenolic acids. The analysis also indicated that the predominant flavonoids in this fraction included rutin (0.94 mg/g), quercetin (0.65 mg/g), and apigenin (0.78 mg/g). Conversely, the samples collected in August showed the presence of methyl gallate (0.29 mg/g), rutin (0.73 mg/g), and apigenin (1.76 mg/g).

The findings indicate a greater abundance and concentration of phenolic and flavonoid compounds in dried *P. guajava* leaves which harvested in March compared to those collected in August. This variation highlights the significant impact of harvest timing on the levels of phytoconstituents, as demonstrated by the fluctuations in phenolic and flavonoid concentrations over time^57,58^.

**Fig. 2:**
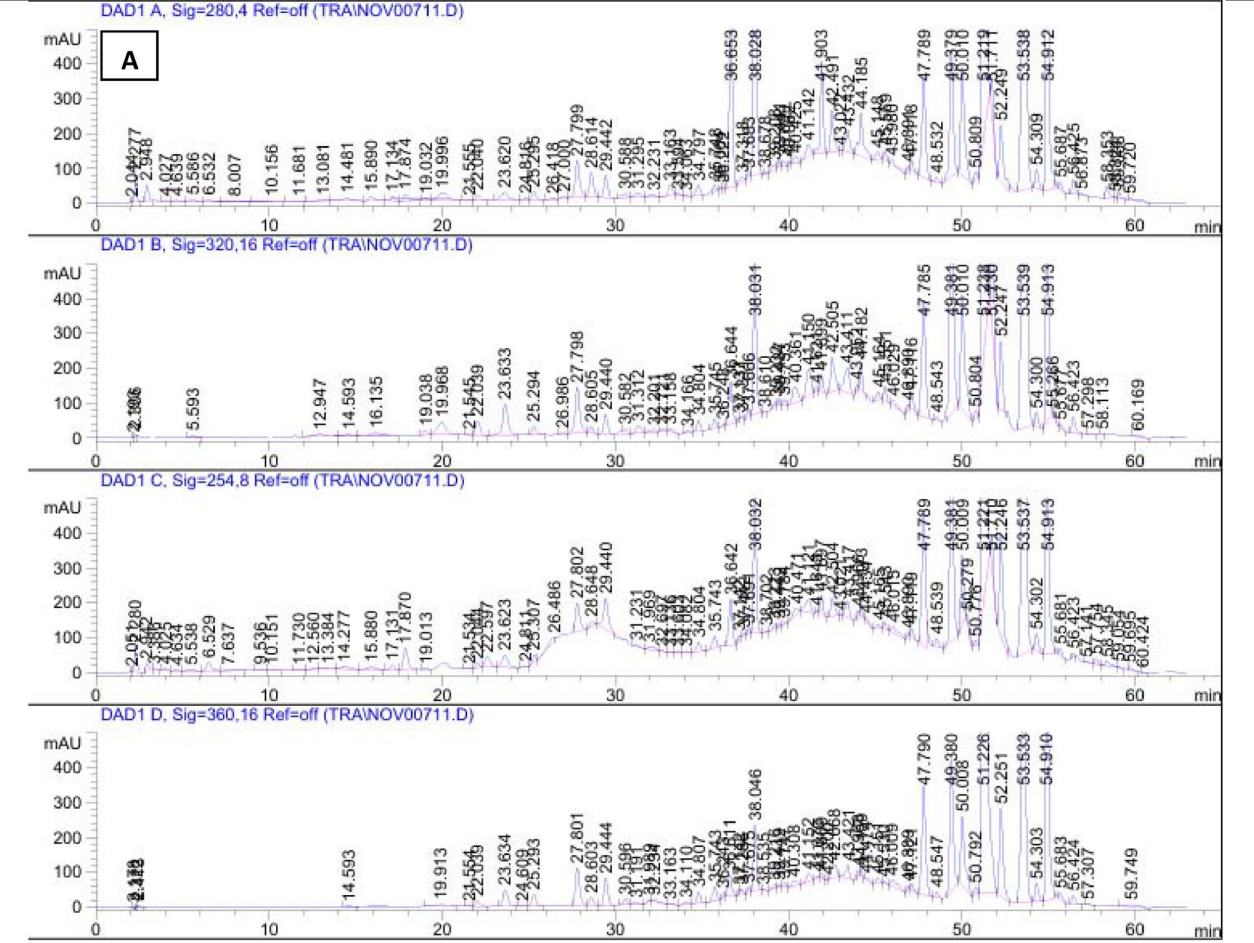

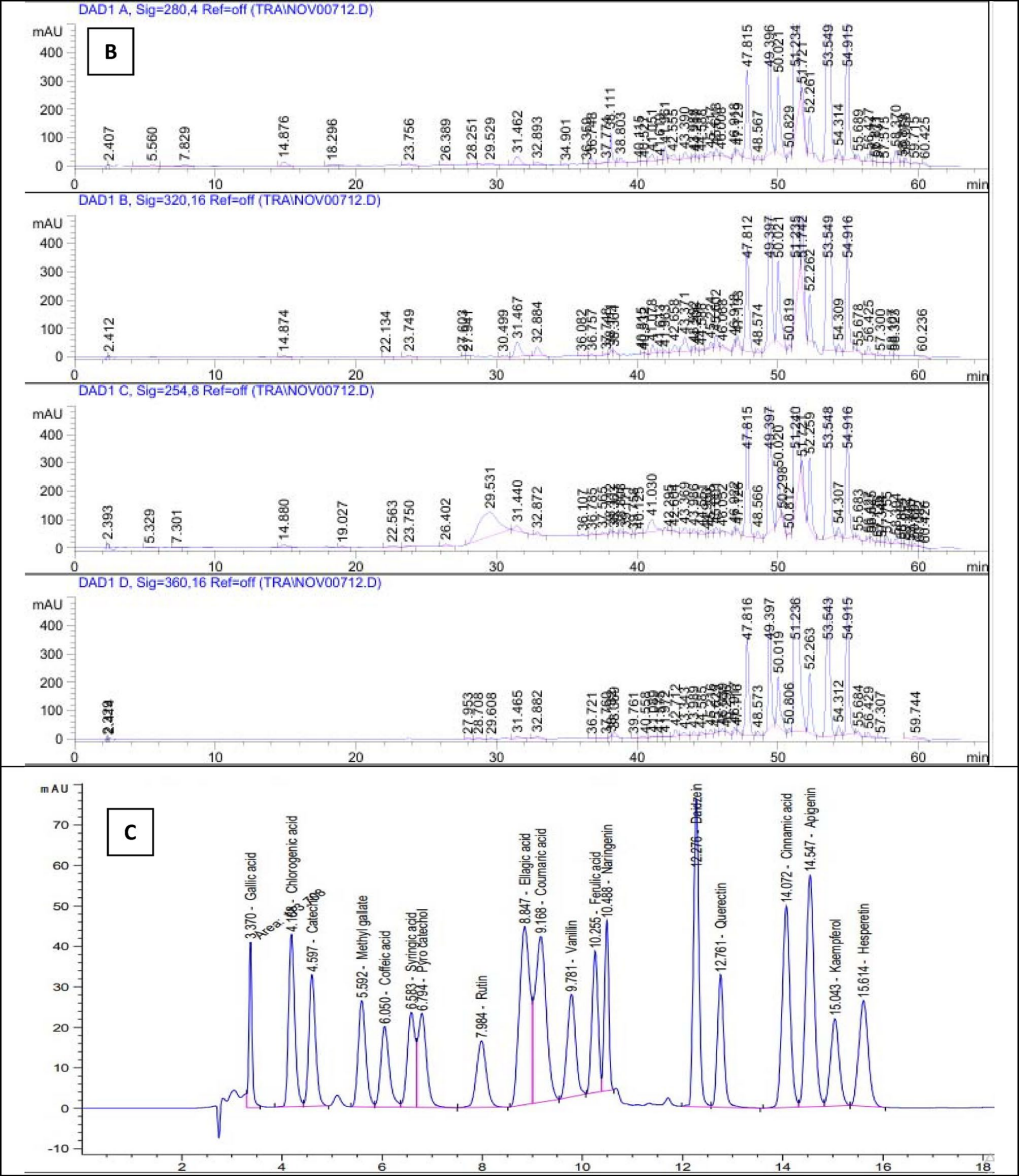
HPLC chromatogram of the ethyl acetate fraction of dried *P. guajava* leaves which collected in March (**A**) and in August (**B**) against standards (**C**).

The findings are consistent with those of Díaz-de-Cerio et al.^59^, who reported that quercetin constitutes a key bioactive phenolic compound in *P. guajava* leaves. Additionally, Wang et al.^60^ highlighted the presence of various other compounds, including gallic acid, rutin, chlorogenic acid, avicularin, isoquercitrin, and kaempferol. In contrast, another study revealed that catechin (2.25%) and epicatechin (1.45%) were found in greater concentrations, whereas lower amounts of gallic acid, chlorogenic acid, quercetin, caffeic acid, and epigallocatechin gallate were detected in *P. guajava* leaf extract^61^. The HPLC chromatograms for the ethyl acetate fraction of *P. guajava* leaves, obtained in both March and August, are illustrated in Fig. 2.

**Table 6:** HPLC analysis for phenolic compounds and flavonoids identified in Ethyl acetate fraction of dried *P. guajava* leaves collected in two different harvest times.

No	Compound	*R*_t_ (min.)	Concentration(mg/g)	
			March	August
1	Gallic acid	3.370	0.24	00
2	Chlorogenic acid	4.168	0.13	00
3	Catechin	4.597	0.56	00
4	Methyl gallate	5.592	0.32	0.29
5	Caffeic acid	6.050	0.21	00
6	Syringic acid	6.583	0.43	00
7	Pyrochatechol	6.794	00	00
8	Rutin	7.984	0.94	0.73
9	Ellagic acid	8.847	00	00
10	Coumaric acid	9.168	0.25	00
11	vanillin	9.781	0.15	00
12	Ferulic acid	10.255	0.17	00
13	Naringenin	10.488	0.03	00
14	Daidzein	12.276	0.12	00
15	Quercetin	12.761	0.65	00
16	Cinnamic acid	14.072	00	00
17	Apigenin	14.547	0.78	1.76
18	Kaempferol	15.043	00	00
19	Hesperitin	15.614	0.51	00

### Characterization of the isolated compounds

The thin-layer chromatographic investigation of extracts from dried *P. guajava* leaves, collected in March and August, led to the identification of six compounds (1–6). The compounds quercetin (1), gallocatechin (2), and kaempferol 7-*O*-glucoside (3) were identified by comparing their Co-TLC profiles with those of known authentic standards. The structural elucidation of morin (4), quercetin 3-*O*-arabinoside (5), and morin-3-*O*-araboinoside (6) was achieved using ^1^H-NMR and ^13^C-NMR techniques. The characterization of compounds 4, 5, and 6 is described as follow:

Compound (4); Morin was obtained as a yellow powder of melting point 300 °C, 31 mg, R_f_=0.79, ^1^H-NMR (400 MHz, CD_3_OD-d4): 6.41 (1H, d, J=2.7 Hz, H-6), 6.23 (1H, d, J=2.7 Hz, H-8), 7.12 (1H, s, H-3’), 6.85 (1H, d, J=7.6 Hz, H-5’), 7.60 (1H, d, J=7.6 Hz, H-6’). ^13^C-NMR (125 MHz, CD_3_OD-d4): 155.32 (C-2), 133.47 (C-3), 177.81 (C-4), 161.96 (C-5), 97.95 (C-6), 164.98 (C-7), 92.87 (C-8), 157.47 (C-9), 104.86 (C-10), 122.11 (C-1’), 145.61 (C-2’), 115.37 (C-3’), 149.10 (C-4’), 114.96 (C-5’), 121.65 (C-6’). The isolated compound, which had been identified earlier, demonstrated results that were consistent with the documentation provided by Tachakittirungrod et al.^62^.

Compound (5); Quercetin 3-*O*-arabinoside was isolated as yellow needles, 24.5 mg, m.p. 255ͦ C; R_*f*_=0.72, ^1^H-NMR (400 MHz, CD_3_OD-d4): 6.42 (1H, d, *J*=2.4 Hz, H-6), 6.20 (1H, d, *J*=2.4 Hz, H-8), 7.69 (1H, s, H-2`), 6.73 (1H, d, *J*=7.9 Hz, H-5`), 7.61 (1H, d, *J*=7.9 Hz, H-6`), 5.23 (1H, d, *J*=7.6 Hz, H-1``), 3.76 (1H, d, *J*=8.7 Hz, H-2``), 3.59 (1H, d, *J*=2.9 Hz, H-3``), 3.77 (1H, m, H-4``), 3.90 (2 H, m, H-5``); ^13^C-NMR (125 MHz, CD_3_OD-d4): 162.35 (C-2), 135.47 (C-3), 181.36 (C-4), 159.03 (C-5), 100.21 (C-6),162.48 (C-7), 94.14 (C-8), 105.35 (C-9), 158.46 (C-10), 121.85 (C-1`), 114.71 (C-2`), 143.93 (C-3`), 151.24 (C-4`), 117.26 (C-5`), 122.43 (C-6`), 104.58 (C-1``), 72.84 (C-2``), 72.05 (C-3``), 69.02 (C-4``), 66.11 (C-5``). The findings associated with the previously identified isolated compound were in agreement with those outlined in the literature by Metwally et al., ^63^.

Compound (6); Morin-3-*O*-arabinoside was isolated as yellow amorphous powder, 27 mg, m.p. 260 ͦ C; R_*f*_= 68, ^1^H-NMR (400 MHz, CD_3_OD-d4): 6.39 (1H, d, *J*=2.9 Hz, H-6), 6.18 (1H, d, *J*=2.9 Hz, H-8), 7.09 (1H, s,H-3`), 6.81 (1H, d, *J*=8.4 Hz, H-5`), 7.57 (1H, d, *J*=8.4 Hz, H-6`), 5.19 (1H, d, *J*=7.8 Hz, H-1``), 3.90 (1H, d, *J*=9.2 Hz, H-2``), 3.64 (1H, d, *J*=3.4 Hz, H-3``), 3.79 (1H, m, H-4``), 3.84 (2 H, m, H-5``). ^13^C-NMR (125 MHz, CD_3_OD-d4): 158.41 (C-2), 133.59 (C-3), 178.74 (C-4), 162.43 (C-5), 98.67 (C-6), 165.48 (C-7), 93.06 (C-8), 158.36 (C-9), 105.14 (C-10), 122.15(C-1’), 146.32 (C-2’), 115.36 (C-3’), 149.52 (C-4’), 115.01 (C-5’), 122.03 (C-6’), 102.97 (C-1``), 72.85 (C-2``), 71.64 (C-3``), 68.43 (C-4``), 65.81 (C-5``). The isolated compound, identified in prior research, showed results that were consistent with the findings reported by Arima and Danno ^64^.

The compounds quercetin (3,3′,4′,5,7-Pentahydroxy flavone) and gallocatechin (Flavan-3,3′,4′,5,5′,7-hexol) were previously detected in the leaves of *Psidium guajava*, as reported by Sampath et al.^65^. Moreover, Kaempherol 7-*O*-glucoside was isolated by Batubara et al.^66^. Quercetin has been documented to exhibit antioxidant, anti-inflammatory, and anti-allergic effects^67^. Gallocatechin is recognized for its antioxidant and anti-cancer properties^68^. Furthermore, kaempherol 7-*O*-glucoside has also been associated with antioxidant and anti-inflammatory activities^69^.

Upon examining the two extracts from dried *P. guajava* leaves, it was determined that gallocatechin (2) and kaempferol 7-*O*-glucoside (3) were present in the ethyl acetate extract from samples collected in both March and August. However, the compounds quercetin (1), morin (4), quercetin 3-*O*-arabinoside (5), and morin-3-*O*-arabinoside (6) were identified solely in the extract from the leaves collected in March.

### Biological investigation

The dried leaves of *P. guajava* contain phytochemical constituents that exhibit diverse biological activities, including antioxidant, hypoglycemic, and anticancer effects, among others^43^. Notably, phenolic and flavonoid compounds are pivotal in regulating various physiological functions, such as cell proliferation, enzymatic processes, cellular redox balance, and signal transduction pathways, thereby playing a significant role in the prevention of chronic diseases^52,70^.

1. Free radical scavenging activity

The production of free radicals during metabolic processes is a critical factor in the emergence of various health problems within the human body. These problems range from inflammatory diseases and ischemic disorders to neurological conditions, hemochromatosis, emphysema, acquired immunodeficiency syndrome, and several other medical issues^71^.

A comparative analysis of the free radical scavenging activity of the ethyl acetate fraction from dried *P. guajava* leaves collected in March and August was performed against DPPH and ABTS radicals at serial concentrations of 10, 50, and 100 µg, as shown in Table 7. The findings revealed that the ethyl acetate fraction from the March collection demonstrated enhanced scavenging capabilities, with recorded values of 69.84 ± 0.20 and 72.16 ± 0.06 for DPPH and ABTS radicals, respectively, at 10 µg/ml. while, at concentration of 50 µg/ml, the values rose to 78.53 ± 0.12 and 78.96 ± 0.13, and at 100 µg/ml, they reached 87.30 ± 0.21 and 83.12 ± 0.09. These results were markedly higher than those obtained from the August collection. The observed concentration-dependent effects are attributed to the higher concentrations of phenolic and flavonoid compounds found in the leaves harvested during the spring.

The antioxidant properties of dried *P. guajava* leaves can be attributed to the presence of various phenolic compounds, including chlorogenic acid, caffeic acid, gallic acid, protocatechuic acid, and ferulic acid, among others^9^. In addition to flavonoids such as rutin, quercetin, apigenin, and kaempferol^72^. Numerous studies have highlighted the antioxidant, anticancer, antidiabetic, and anti-inflammatory properties of flavonoids^29,73^. The effectiveness of flavonoids is closely linked to their molecular structure, particularly the number and positioning of hydroxyl groups, as well as the conjugation and resonance characteristics^74,75^.

The role of antioxidant compounds found in *P. guajava* leaves in mitigating the adverse effects of free radicals has been substantiated by a variety of research studies. Chen and Yen^5^ identified a linear relationship between the potency of antioxidants, their capacity to neutralize free radicals, and the phenolic content in *P. guajava* leaves. In addition, a subsequent study demonstrated the synthesis of silver nanoparticles from crude polysaccharides of *P. guajava* leaves, which exhibited remarkable scavenging activities against DPPH radicals and ABTS radical cations^60^. These results indicate that *P. guajava* leaf extracts could be valuable as antioxidant resources in both food preservation and cosmetic applications.

**Table 7:** The free radicals scavenging activity of Ethyl acetate fraction of dried *P. guajava* leaves collected in two different harvest times against DPPH and ABTS.

Ethyl acetate fraction	DPPH				ABTS			
	Inhibition percentages (%)			IC50	Inhibition percentages (%)			IC50
	10 µg/ml	50 µg/ml	100 µg/ml		10 µg/ml	50 µg/ml	100 µg/ml	
In March	69.84 ± 0.20^a^	78.53 ± 0.12^b^	87.30 ± 0.21^c^	25.90	72.16 ± 0.06^a^	78.96 ± 0.13^b^	83.12 ± 0.09^c^	25.30
In August	65.98 ± 0.16^d^	74.11 ± 0.08^e^	81.27 ± 0.12^f^	30.89	70.48 ± 0.13^d^	76.03 ± 0.06^e^	79.98 ± 0.10^f^	28.079
Ascorbic acid	73.12 ± 0.15^g^	82.47 ± 0.13^h^	90.52 ± 0.09^i^	22.40	76.02 ± 0.11^g^	81.43 ± 0.08^h^	87.26 ± 0.13^i^	21.613

Values are represented by mean ± SD of three replicate. The different superscript letters between groups are significant differences at P values < 0.05.

#### In vitro antidiabetic activity

Diabetes represents a significant chronic health issue, affecting approximately 10% of the global population with a disorder of blood glucose metabolism, primarily marked by a state of hyperglycemia. This condition can arise from either a deficiency in insulin production by the β-cells of the pancreatic islets, as seen in type 1 diabetes, or from the cells’ failure to respond adequately to the insulin that is secreted, characteristic of type 2 diabetes^76^. The sustained hyperglycemic state results in overproduction of reactive oxygen species (ROS) and dyslipidemia, leading to significant cellular damage and a range of complications^77^.

The in vitro assessment of the antidiabetic activity of the ethyl acetate fraction from dried *P. guajava* leaves was conducted using three varying concentrations (10, 50, and 100 µg/mL). The results, detailed in Table 8, reveal that the ethyl acetate extract demonstrated significant inhibitory activity against carbohydrate-metabolizing enzymes, including α-amylase, α-glucosidase, and β-galactosidase, in a dose-dependent manner. Notably, the antidiabetic effects were found to be more substantial in dried *P. guajava* leaves which harvested in March than in those collected in August. This observation highlights the critical role of harvesting time in influencing the phytochemical profiles and biological properties of *P. guajava* leaves.

The bioactive constituents found in dried *P. guajava* leaves have demonstrated efficacy in mitigating the risk of diabetes. Several studies have documented the antidiabetic effects of flavonoids derived from *P. guajava* leaves, reporting substantial improvements in the functionality of β-cells in pancreatic islets and the morphology of hepatocytes in diabetic mice^78,79^. Furthermore, research focusing on the hypoglycemic properties of dried *P. guajava* leaf extract has indicated that its phenolic compounds can enhance vascular function in mice with obesity induced by diet^80^.

A different study assessing the food-drug interactions of *P. guajava* leaf tea found no potential interactions with medications, thereby supporting the conclusion that guava leaf tea is safe regarding food-drug interactions. Individuals with borderline diabetes often consume guava leaf tea to control the swift rise in blood sugar levels post-meal, as the tea is rich in carbohydrates and dietary polyphenols that bind to digestive enzymes, which are believed to promote health by limiting the absorption of dietary sugars and lipids^81^. Furthermore, a recent analysis of herbal teas also confirmed that guava tea does not exhibit any interactions with pharmaceuticals^82^.

**Table 8:** Inhibitory activity of carbohydrate metabolizing enzymes by the Ethyl acetate fraction of dried *P. guajava* leaves which collected in two different harvest times.

α-amylase						
Concentration (µg /mL)	In March		In August		Acarbose	
	Inhibition %	IC50	Inhibition %	IC50	Inhibition %	IC50
**10**	39.34 ± 0.14^a^	20.57	34.37 ± 0.14^b^	33.15	38.57 ± 0.17^e^	18.057
**50**	73.86 ± 0.08^c^		67.02 ± 0.07^e^		79.54 ± 0.12^d^	
**100**	80.97 ± 0.11^d^		75.73 ± 0.20^c^		86.31 ± 0.09^f^	
**α-glucosidase**						
**Concentration** **(µg /mL)**	**In March**		**In August**		**Acarbose**	
	**Inhibition %**	**IC50**	**Inhibition %**	**IC50**	**Inhibition %**	**IC50**
**10**	38.37 ± 0.13^e^	19.54	32.01 ± 0.06^a^	31.89	41.89 ± 0.10^c^	13.37
**50**	77.46 ± 0.16^c^		71.36 ± 0.10^c^		80.36 ± 0.08^d^	
**100**	83.91 ± 0.14^f^		79.89 ± 0.21^d^		89.57 ± 0.15^f^	
**β- galactosidase**						
**Concentration** **(µg /mL)**	**In March**		**In August**		**Acarbose**	
	**Inhibition %**	**IC50**	**Inhibition %**	**IC50**	**Inhibition %**	**IC50**
**10**	41.94 ± 0.11^a^	14.52	37.84 ± 0.13^b^	34.69	43.78 ± 0.12^e^	8.63
**50**	78.62 ± 0.06^c^		70.98 ± 0.20^c^		83.72 ± 0.09^f^	
**100**	89.53 ± 0.18^f^		82.04 ± 0.07^d^		91.40 ± 0.10^g^	

Enzymes are expressed as %. Data are mean ± SD of 3 replicates. Statistical analysis is carried out using one way analysis of variance (ANOVA), combined with post hoc and Co-Stat computer program, where different superscript letters between groups are significant differences at P values < 0.05.

#### In vitro antiinflammatory evaluation

Cyclooxygenase-2 (COX-2) and lipoxygenase (5-LOX) are recognized as significant pro-inflammatory enzymes that play a crucial role in the metabolism of arachidonic acid^83^. In recent years, there has been considerable interest among researchers in developing selective inhibitors for COX-2 and 5-LOX, aiming to mitigate the adverse effects associated with non-steroidal anti-inflammatory drugs (NSAIDs) and to explore novel therapeutic strategies for various conditions, including cancer and chronic inflammatory diseases^84^. This study aims to identify a dual inhibitor of COX-2 and 5-LOX by examining the anti-cyclooxygenase and anti-lipoxygenase activities of dried *P. guajava* leaves through an enzymatic assay. The results, including percentage inhibition and IC50 values for two ethyl acetate fractions of dried *P. guajava* leaves which collected in March and August, alongside reference drugs, are detailed in Table 9.

It was found that the ethyl acetate fraction from dried *P. guajava* leaves which collected in March showed a superior inhibition of COX-2 enzyme activity (Inhibition 78.34 ± 0.17%; IC50 3.95 ± 0.14 µg/mL) and 5-LOX enzyme activity (Inhibition 68.11 ± 0.12%; IC50 6.23 ± 0.10 µg/mL) relative to *P. guajava* extract gathered in August. This was evaluated against the standard medications Indomethacin and Zileuton.

The anti-inflammatory properties of dried *P. guajava* leaves can be attributed to their significant flavonoid content. Flavonoids are characterized by a benzene ring (A) that is fused with a six-membered ring (C) and features a phenyl ring (B) at the 2-position^85^. Research indicates that these compounds exhibit anti-inflammatory effects during both the proliferative and exudative stages of inflammation by inhibiting various enzymes, including nitric oxide synthase, xanthine oxidase, lipoxygenase (LOX), and cyclooxygenase (COX)^86^. The proposed mechanism of action involves the formation of hydrogen bonds with the active site of COX enzymes, as well as π–π interactions between the phenyl ring of the flavonoid and the Tyr355 residue at the entrance of the COX binding site. This binding is further enhanced by hydrophobic interactions within the hydrophobic and lobby regions of the COX enzyme’s binding site. In the case of 5-LOX inhibition, the inhibitory effect is attributed to hydrogen bonding with the Ala424 residue^87^.

**Table 9:** In vitro anti-inflammatory activities of the Ethyl acetate fraction of dried *P. guajava* leaves collected in two different harvest times using different concentrations (1,10,100 µg).

Ethyl acetate fraction	COX-2				5LOX			
	**Inhibition (**%**)**			**IC50**	**Inhibition (**%**)**			**IC50**
	**1 µg/ml**	**10 µg/ml**	**100 µg/ml**		**1 µg/ml**	**10 µg/ml**	**100 µg/ml**	
In March	45.61 ± 0.18 ^a^	52.06 ± 0.19 ^a^	78.34 ± 0.17^a^	9.47	39.12 ± 0.14 ^f^	61.43 ± 0.07 ^e^	68.11 ± 0.12^b^	6.54
In August	39.87 ± 0.12 ^b^	55.48 ± 0.21^g^	71.56 ± 0.08^e^	15.54	37.84 ± 0.11 ^b^	54.82 ± 0.10 ^d^	64.05 ± 0.09^d^	25.76
Indomethacin	40.29 ± 0.09 ^d^	59.18 ± 0.17 ^a^	84.37 ± 0.16^c^	7.18				
Zileuton					40.62 ± 0.08 ^e^	61.39 ± 0.14 ^f^	79.42 ± 0.13^f^	3.66

Data are mean ± SD of 3 replicates, different superscript letters between groups are significant differences at P values < 0.05.

## Conclusion

*Psidium guajava* leaves are deemed a promising reservoir of natural compounds that are easily accessible. Their significant levels of minerals and vitamins encourage their use as a direct source of nutrition. Furthermore, *P. guajava* leaves contain a variety of secondary metabolites, including flavonoids and other phenolic compounds. These phytochemicals have the potential to enhance and stabilize various physiological and metabolic processes within the human body, serving as important immune stimulators and modulators for chronic conditions such as diabetes, cancer, and diseases affecting the gastrointestinal, neurodegenerative, and cardiovascular systems.

The investigation of phyto-constituents in *P. guajava* leaves revealed significant differences in their composition and concentration, which were influenced by the timing of harvest and the relationship between soil and plant. Specifically, *P. guajava* leaves harvested in March exhibited higher phytoconstituents concentrations than those collected in August. These results highlight the importance of harvest timing in assessing phytochemical content and biological effects, indicating that this variable must be taken into account during the sampling of *P. guajava* leaves. Additionally, careful monitoring of soil nutrient deficiencies is vital for optimizing *P. guajava* yield.

## Data Availability

The datasets used and/or analysed during the current study available from the corresponding author on reasonable request.
